# Motivation for COVID-19 vaccination among international students in Russia

**DOI:** 10.1192/j.eurpsy.2022.1371

**Published:** 2022-09-01

**Authors:** E. Nikolaev, S. Petunova

**Affiliations:** Ulianov Chuvash State University, Social And Clinical Psychology Department, Cheboksary, Russian Federation

**Keywords:** COVID-19 vaccination, motivation, international students

## Abstract

**Introduction:**

After Russia decided to start COVID-19 vaccination of international students who are getting their education on its territory, they received an opportunity to get a single dose of COVID-19 Sputnik Light vaccine. What motives can such international students have for being vaccinated in the situation of uncertainty?

**Objectives:**

Our goal is to define the structure of motivation for COVID-19 vaccination among international students who are getting education at different departments of the university.

**Methods:**

In October 2021, we surveyed 409 international students getting education at Ulianov Chuvash State University in Cheboksary, who agreed to COVID-19 vaccination.

**Results:**

Those who applied for vaccination were mostly 3^rd^ year students (32.03%) and 4^th^ year ones (21.52%). 8 students out of the surveyed (1.96%) had been vaccinated outside Russia, 4 – in Russia (0.98%). 8.56% of the pool had had COVID-19, 57.7% had not, 33.74% could not give a certain answer. Main motives for COVID-19 vaccination were: unwillingness to be ill (57.21%), unwillingness to have any limitations imposed (22.98%), unwillingness, especially of medical students, to have problems in their studies (12.22%), inclination towards following their relatives’ advice (5.13%), desire to follow the surrounding people’s example (1.98%). In personal conversations, the students often expressed their wish for being vaccinated with a 2-component Sputnik V vaccine for a better protection from illness.

**Conclusions:**

The survey of the international students’ motives showed that most of them have positive attitude to COVID-19 vaccination and feel inclined to be vaccinated with a Russian vaccine in order to reduce the risk of getting ill.

**Disclosure:**

No significant relationships.

